# Acute kidney injury in critically ill patients with A/H1N1 pneumonitis in 2010/11

**DOI:** 10.1186/cc10952

**Published:** 2012-03-20

**Authors:** M Atkinson, A Krige, S Chukkambotla

**Affiliations:** 1 East Lancashire Hospitals NHS Trust, Blackburn, UK

## Introduction

A/H1N1 infection is a major seasonal cause of illness requiring critical care admission. A high proportion of these patients develop acute kidney injury (AKI) [1].

## Methods

We studied all A/H1N1-positive admissions to a district general hospital (DGH) ICU during the months of December 2010 and January 2011. The study aimed to describe the incidence of AKI using the creatinine score from the RIFLE criteria and its associations with mortality, incidence and duration of intermittent positive pressure ventilation (IPPV), length of stay in the ICU and provision of renal replacement therapy (RRT).

## Results

Twenty-seven patients were admitted to the ICU who tested positive for A/H1N1. Fourteen (52%) met the RIFLE criteria for AKI. Of these, three (11%) met the RIFLE criterion for Risk (>150% change in creatinine), three (11%) met the criterion for Injury (>200% change in creatinine), and eight (30%) met the criterion for Failure (>300% change in creatinine). Nine patients (33% of all patients, 64% of AKI patients) received RRT. ICU mortality was three out of 14 (21%) patients with AKI and one out of 13 (8%) patients without AKI. This difference was not statistically significant. Thirteen out of 20 (65%) ventilated patients developed AKI, compared with one out of seven (14%) nonventilated patients. This difference was statistically significant (*P *= 0.0329). Excluding fatalities, the duration of IPPV was longer in patients with AKI (median 11 days, range 0 to 54 days) than in patients without AKI (median 1 day, range 0 to 20 days). This difference was statistically significant (*P *< 0.05). Excluding fatalities, the length of stay was longer in patients with AKI (median 19 days, range 10 to 68 days) than in patients without AKI (median 5 days, range 2 to 29 days). This difference was statistically significant (*P *< 0.02).

## Conclusion

We noted a higher incidence of AKI in critical illness associated with A/H1N1 (52%) compared to that of a larger study [1]. AKI was associated with the incidence as well as duration of mechanical ventilation and length of stay in the ICU. The use of RRT in the current study (60%) was much higher than in the modeling study (16%). We found a trend towards greater mortality with AKI, although (unlike Petillä and colleagues [1]) this failed to reach significance.
